# Intensive care for human hearts in pluripotent stem cell models

**DOI:** 10.1038/s41536-020-0090-7

**Published:** 2020-03-06

**Authors:** Pelin Golforoush, Michael D. Schneider

**Affiliations:** National Heart and Lung Institute, Imperial College London, London, W12 0NN UK

**Keywords:** Stem-cell biotechnology, Drug development

## Abstract

Successful drug discovery is ultimately contingent on the availability of workable, relevant, predictive model systems. Conversely, for cardiac muscle, the lack of human preclinical models to inform target validation and compound development has likely contributed to the perennial problem of clinical trial failures, despite encouraging non-human results. By contrast, human cardiomyocytes produced from pluripotent stem cell models have recently been applied to safety pharmacology, phenotypic screening, target validation and high-throughput assays, facilitating cardiac drug discovery. Here, we review the impact of human pluripotent stem cell models in cardiac drug discovery, discussing the range of applications, readouts, and disease models employed, along with the challenges and prospects to advance this fruitful mode of research further.

## The unmet need: resuscitating cardiac drug discovery

Notwithstanding decades of aggressive risk factor reduction, as well as transforming health care delivery systems to restore coronary flow urgently in myocardial infarction, the prevalence of heart disease remains predominant—wholly unchanged in rank as the single leading cause of death and disability for both men and women worldwide^1,2^. Ischemic heart disease remains, within this, the preponderant form^1,2^. From 2007 through 2017, the age-adjusted death rates from ischemic heart disease fell 9.7%, which is gratifying progress, yet to focus on this one metric would be quite misleading. Indeed, during this same period, the actual number of deaths from ischemic heart disease increased 22.3%, and years of life lost increased to the same degree^2^. Similar concerns arise from the adverse trends in “disability-adjusted life-years,” a measure of healthy life expectancy^1^. In short, progress in allaying ischemic heart disease is stymied, at the epidemiological level, and the burden—to patients, their families, care-givers, and health care systems—remains stupefying. The pandemic continues, unabated.

**Fig. 1:**
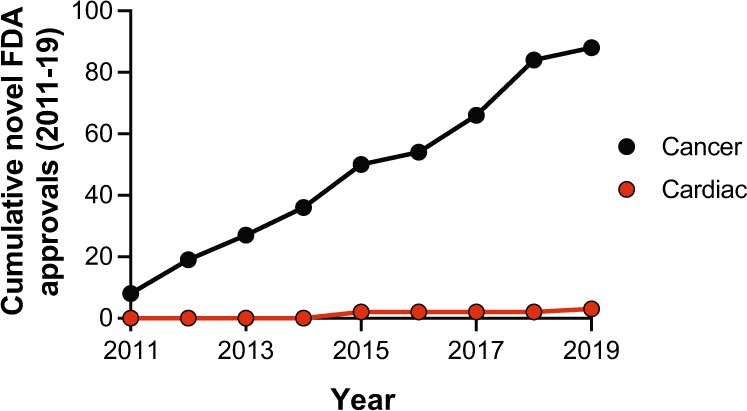
Lost decade: a moribund cardiac drug discovery pipeline. Shown, by year, are the cumulative US Food and Drug Administration NME approvals for cancer chemotherapy (consistently, 20–25% of the total approvals each year), compared with the paucity of new drugs targeting cardiac muscle^110^. See text for details.

In this context, far from subsiding, the need remains paramount for novel, transformative therapies to mitigate heart muscle cell death and dysfunction. However, as measured by the number of new cardiac drugs that enter clinical practice, making innovation workable has shown dismaying lack of success^3,4^. From 2011 to 2019, the US Food and Drug Administration approved nearly 90 novel drugs for cancer but, astoundingly, a mere four that target cardiac muscle directly (Fig. 1): sacubitril/valsartan for heart failure^5^, ivabradine for heart failure^6,7^, deferiprone for transfusional iron overload in thalassemia^8^, and tafamidis meglumine for cardiomyopathy in transthyretin amyloidosis^9^. (Additional drugs approved for broader cardiac indications were those for which cardiac muscle is neither the mechanistic focus of disease nor the site of the corresponding benefit, such as anti-platelet agents, Factor Xa inhibitors, and anti-hyperlipidemics.)

Many drugs fail to reach approval for reasons of efficacy, and in the realm of cardioprotection—seeking to rescue jeopardized human heart muscle beyond the benefits of reperfusion alone—such failures have been rife. Time and again, proposed counter-measures, some with highly credible support from whole-animal studies of myocardial infarction, failed to show the anticipated efficacy^10–15^. But, there is a disturbing paucity even of new cardiac drug candidates put forward into early phase development, i.e., the number of New Molecular Entity (NME) applications seeking to initiate human trials^3^. In short, both ends of the cardiac drug pipeline are dry.

What are the barriers to trying more often, more effectively? Which, if any, might be remediable? Among the myriad obstacles most cited are: the drug development costs to bring a new agent to market; regulatory uncertainties; discrepancies in philanthropy and research grant support; the lack of appealing biological targets; commercial viability; the length, size, and complexity of trials required; risks of reliance on surrogate endpoints; and, of course, poor return on investment, given the frequency and cost of clinical failures^3,16^. The net result is, consequently, few novel “first-in-class” therapies. Expert think tank recommendations have focused on the trials eco-system (reducing operating costs, streamlining trial design, embracing adaptive design, funding early-phase development more assiduously) but, appropriately, also call for strengthening “novel scientific methods to further define the pathophysiology”^3^.

One key driver of failure in human cardiac trials is, likely, the recurring lack of systematic human preclinical data for target validation and compound development, i.e., a gap in the information available to de-risk the proposition before ever entering human trials. Model organisms, ranging from the traditional to bespoke genetic lines, are instructive, to be sure, but have, in the aggregate, failed thus far to show sufficient predictive power for efficient translation of targets and drugs to benefit human health^17^. The abysmal track record for cardiac drug development speaks for itself. Drawing again on oncology as an instructive comparison, cancer drugs enter human trials having first, at a very early stage, been vetted in dozens to hundreds of well-characterized human cancer cell lines, available as turnkey resources in laboratories worldwide^17–19^. Highlighted by the National Cancer Institute’s pioneering 60 human tumor cell line screen^20^ and by later, larger initiatives including the Cancer Cell Line Encyclopedia^21,22^, the role played by human cancer cells in cancer drug discovery is central, essential, and incontrovertible. In contrast, human heart muscle has never been available for equivalent proof-of-concept studies, other than sporadically—through biopsies and explanted hearts—never the routinized, scalable, renewable resource required for library screening, systematic compound development, and pull-through to translation. Apart from this want of starting material, even short-term expansion of adult human cardiomyocytes in primary culture is thwarted by the terminally differentiated myocytes’ characteristic state of growth arrest.

**Fig. 2:**
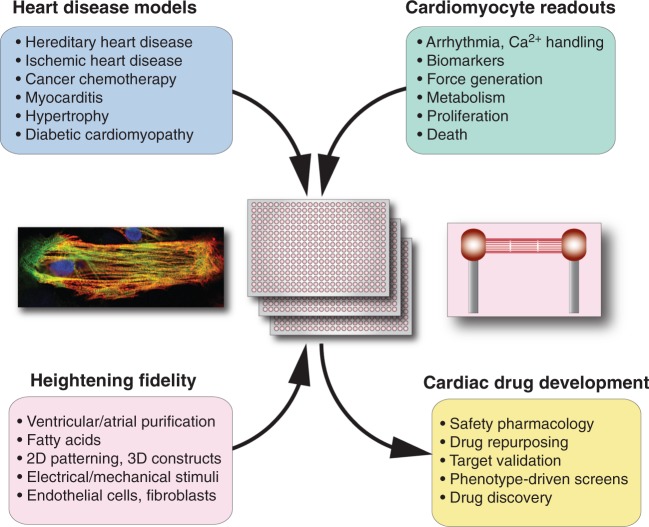
Enhancing cardiac drug discovery in hPSC-CMs. Diverse cardiac disorders have been successfully modeled in hPSC-CMs, beyond merely the patient-specific mutations for which this technology was first used, with broad applicability now demonstrated for the widespread, acquired forms of human heart disease. Concurrently, the readouts relevant to cardiac drug development have expanded beyond the arrhythmias first studied, to encompass the full spectrum of molecular and functional cardiomyocyte phenotypes including mechanical performance, energetics, myocyte formation, and myocyte loss. The impact on drug development has been manifested initially through more predictive safety pharmacology (including the improved profiling of non-cardiac drugs) and through human preclinical studies of approved agents, toward novel applications. In the development of novel agents, hPSC-CMs can augment not only target-based approaches, as platforms for validation by gene silencing and the investigation of new chemical series, but also as a human substrate for mechanistically agnostic, phenotype-driven screens. Diverse approaches promote cardiomyocyte maturation and fidelity to the adult human heart itself, which remains an acknowledged limitation of these models.

By contrast, human cardiac muscle cells made in limitless quantities from human pluripotent stem cells (hPSC-CMs) now provide unprecedented access to “heart disease in a dish,” with encouraging potential to accelerate the present tepid pace of cardiac drug discovery^23–32^ (Fig. 2; Table 1). Cultured from the inner cell mass of the blastocyst, human embryonic stem cells (hESCs) are the native “gold standard” for human pluripotency^33^, have all the requisite properties including efficient cardiomyocyte creation^34^, and have even progressed into primate trials of cardiomyocyte grafting^35^. Human induced pluripotent stem cells (hiPSCs) are made instead from adult somatic cells reprogrammed with ESC transcription factors^36^, augmented or replaced by chemical reprogramming. Compared to hESCs, hiPSCs have similarly well-proven cardiogenic potential^34^ but pose fewer ethical or religious concerns, and make possible the interrogation of patient-specific genetic variants^24,37^, in addition to modeling the pandemic forms of acquired heart disease that have greatest public health significance. Here, except if otherwise noted, we refer to hPSCs, for ESCs and iPSCs collectively.

**Table 1:** Summary of representative studies using hPSC-derived cardiomyocytes to enhance cardiac drug development.

Objective	Design	Readouts	Comments	Refs.
Safety pharmacology	Hoffman-La Roche: 28 compounds	MEAs, impedance	Improved accuracy over hERG screening	^40^
Safety pharmacology	GSK: 10 compounds	MEAs	Concurred with rabbit ventricular wedge	^41^
Safety pharmacology	J&J: 20 compounds	MEAs	90% accuracy for known toxicities	^42^
Safety pharmacology	Quintiles: 24 compounds	Impedance, cell number, cTnI, ROS, lipid acumulation		^106^
Safety pharmacology	AZ: 51 compounds	Intracellular Ca2+, edge detection	87% sensitivity, 70% specificity	^111^
Safety pharmacology	CiPA: 28 compound screen, 10-site study	MEAs, voltage-sensitive dye	Blinded, AUC 0.87	^32^
Safety pharmacology	JiCSA: 60 compounds	MEAs	Marker of risk for onset of Torsade de pointes	^46,47^
Safety pharmacology	CRACK IT InPulse	Contractility, metabolic maturation	Substrate microarrays, EHT	^49^
Safety pharmacology	HER2-targeted liposomal DOX	DOX uptake, viability, apoptosis	Phase 2: no efficacy against breast cancer	^58^
Long QT syndrome	Protein chaperone to fix hERG trafficking	MEAs	Successfully advanced to first-in-human study	^60,61^
Coxsackie B myocarditis	4 known anti-virals	CVB3-luc	CAR expression 30× lower than in adult LV	^62^
Cardiac hypertrophy	ET-1-induced hypertrophy	BNP (hypertrophy), nuclear count (toxicity)	384-well format	^64^
Diabetic cardiomyopathy	Phenotype-driven screen	BNP, sarcomere integrity, Ca^2+^ transients, impedance, electrophysiology	Protective compounds identified	^77^
Cardiotoxicity	Protocols to derive & analyze hPSC-CMs	Imaging viability and contractility	Development of “cardiac safety index”	^54^
Cardiotoxicity	TKI-induced toxicity: 21 compounds	Viability, contractility, Ca^2+^ transients; RTK phosphorylation	Protection by IGF1 or insulin	^26^
Cardiotoxicity	DOX-induced toxicity; *TOP2B* disruption by CRISPR-Cas9	Viability, ΔΨm, ROS, [Ca^2+^]_i_, DNA damage, MEAs, RNA-seq	Role of *TOP2B* substantiated	^53^
Cardiotoxicity	TKI-induced and DOX-induced toxicity	RNA-seq, mass spec, mitochondrial function, metabolomics	TKIs disrupt metabolism; DOX induces DNA damage	^56^
Cardiomyocyte survival	Genetic & small molecule inhibitors of MAP4K4	Viability, apoptosis, Ca^2+^ cycling, mitochondrial function, force generation	Protection in 2D & 3D culture; reduced infarct size in mice	^65^
Cardiomyocyte survival	Phenotype-driven HTS	Viability, expression of cardioprotective genes	HO-1 correlates with myocyte protection	^75^
Cardiomyocyte proliferation	Phenotype-driven HTS	High-throughput proteomics, RNA-seq	Pro-proliferative compounds identified	^84^

## hPSC-derived cardiomyocytes for predictive toxicology: canaries in a coal mine

Historically, the unmet scientific need to consider hPSC-CMs in the context of drug development begins with safety pharmacology: namely, the failure of conventional animal models to predict drug toxicities^38^. In the USA, roughly one in seven approved compounds is later withdrawn from clinical use, 28% of these for adverse cardiac events, including potential lethal arrhythmias, muscle cell death, and heart failure^39^. Using microelectrode arrays (MEA), pioneering studies by Hoffman La-Roche^40^, GlaxoSmithKline^41^ and Johnson and Johnson^42^ profiled 10 to 30 compounds, for their pro-arrhythmic effects in hPSC-CMs. All three surveys concluded that the relevant pharmacology was captured in these human cardiomyocyte assays, with obvious inherent advantages over non-cardiac cells that are engineered to express a single human cardiac ion channel such as hERG (substrate for the ventricular arrhythmia Torsade de Pointes [TdP]). The hPSC-CMs enabled very high accuracy over the relevant range of concentrations, compared to standard lower throughput, higher cost ex vivo methods (ventricular wedge, Purkinje fiber, Langendorff). Indeed, hPSC-CMs were superior to the routine preclinical models for detecting some key parameters of risk, such as effects on repolarization akin to human QTc prolongation^41^.

Building on these encouraging findings, more systematic use of hPSC-CMs has been promoted, together with in silico modeling and other prediction tools, by the Comprehensive In Vitro Proarrhthymia Assessment (CiPA) initiative^25,32,43^. Explicitly, hPSC-CMs are viewed as “more complete ‘biological integrators’ that detect not only complex effects of drugs on multiple cardiac currents, but also modulatory effects on currents elicited through signaling pathways, channel-associated subunits, altered intracellular calcium handling, additional transporters and exchangers, and potential changes in channel densities in myocytes”^25^. The potential utility of hPSC-CMs to predict drug-induced proarrhythmic effects was demonstrated most conclusively in a blinded, international, 10-site study of 28 drugs, using two commercially available lines and diverse electrophysiological platforms including MEAs and voltage-sensing potentiometric dyes^32^. The test compounds were first categorized by degree of known clinical risk for TdP, then were analyzed in blinded fashion for the prevalence of drug-induced repolarization abnormalities and arrhythmia-like events. These data, from 15 cell type × platform combinations, were then used to a construct a predictive model of TdP risk. Significant predictors in the hPSC-CMs were: arrhythmia-like event at any tested concentration, maximum prolongation at any tested concentration, and estimated prolongation at the clinical concentration of drug. These parameters in turn were fed into a composite model of TdP risk, with dichotomous outcomes (low, versus high or intermediate). The receiver operating characteristic (ROC) area under the curve (AUC) was 0.87, regardless of the cell line used or any local differences in culture method. In short, blinded studies have made it clear that measurements in hPSC-CMs are a highly reliable preclinical classifier of clinical TdP risk. Given such evidence, hiPSC-CMs have gained acceptance by industry and regulatory authorities as predictive of drug safety in humans, at least with respect to arrhythmic risk ^25,32,44,45^.

Two further initiatives with hPSC-CMs for safety pharmacology are the Japan iPS Cardiac Safety Assessment (JiCSA)^46,47^ and the CRACK IT InPulse Challenge^48–50^. Key lessons from JiCSA, which likewise is focused on arrhythmic risk, have included fidelity of the relationship between MEA-measured field potential duration and interspike interval in hPSC-CMs to the QT-RR relation deduced from clinical electrocardiograms in the Framingham Heart Study^46^. Human-relevant characteristics included repolarization delay at slow beating rates, reverse use-dependency, categorical analyses as potential indices of risk, and a threshold field potential duration that predicts early afterdepolarizations (EADs) and triggered activity, useful as a potential marker of risk for the onset of human Torsade de pointes^46^. A subsequent large-scale validation study of 60 compounds’ torsadogenic risk markedly expanded the conclusions available from CiPA^32^, though not yet in blinded fashion. Findings were highly concordant in iCell and Cor.4U hPSC-CMs^47^, despite the lines’ differences in ion channel and membrane transporter expression ^51^.

The InPulse academic-industry consortium, by contrast, has emphasized developing a robust in vitro platform to monitor cardiac contractility, with cells that are phenotypically mature^48–50^. (See the Challenges and Prospects section for a detailed discussion of this concern.) This project’s discoveries include a synthetic polymer that promotes the maturity of hPSC-CMs (a co-polymer of isobornyl methacrylate and *tert*-butylamino-ethyl methacrylate), identified using combinatorial subtrate material microarrays^48^, novel open-source tools for quantifying cardiomyocyte contraction^49^, and proof that driving contractile work in hPSC-CMs induces mitochondrial biogenesis and recapitulates the normal post-natal shift to fatty acid oxidation ^50^.

Other cardinal features of potential cardiotoxicity—notably, myocyte loss and ensuing heart failure—are likewise amenable to profiling in hPSC-CMs, with the cardiotoxicity of anti-cancer drugs being an especially robust area of investigation^26,52–54^. Readily applicable assays include biochemical readouts (defective ATP production, cardiac troponin release), microscopy (loss of surface membrane integrity, apoptosis), and video imaging to assess contractility (edge detection or deformation maps). As was done for arrhythmic risk, these quantitative parameters of toxicity can be integrated as a composite cardiac safety index^26,54^. Interestingly, interpatient variations in heart tissue transcriptomics were largely recapitulated in patient-specific hPSC-CMs, including the *NRF2*-*PPARGC1A* pathway controlling oxidative stress, and correlated well with the patient-derived cells’ functional difference in cardiotoxic responses^31^. In a related study, patient-specific hPSC-CMs reproduced the patients’ respective vulnerability to trastuzumab^55^. More recently, a network-level comparison of cardiotoxicity in hPSC-CMs used RNA sequencing to distinguish differences in the pathways engaged by anthracyclines versus tyrosine kinase inhibitors^56^. Here, the principal finding was the categorically different transcriptomic signatures evoked by these anti-cancer agents in human cardiomyocytes. Specifically, whereas doxorubicin (DOX) induces pathways that initiate DNA damage, tyrosine kinase inhibitors disrupt mitochondrial energetics even at non-lethal concentrations, downregulating oxidative phosphorylation and upregulating glycolysis^56^, metabolic reprogramming that is a common feature of hypertrophied and failing hearts^57^. In agreement with DNA damage as the main target for DOX, genome editing to delete topoisomerase-II beta (TOP2B) markedly reduced the vulnerability of hPSC-CMs to DOX-induced DNA double strand breaks and cell death ^53^.

## HPSC-CMS in cardiac drug development: early exemplars

As a potential step change beyond just safety assessment and risk prediction, might hPSC-CMs also be useful as human preclinical proof of efficacy, to guide and inform experimental therapeutics? In one early study of this kind, investigators sought to mitigate the known cardiotoxicity of DOX, a mainstay of cancer chemotherapy, as mentioned, targeting DOX selectively to breast cancer using liposomes conjugated with antibody against human epidermal growth factor receptor 2 (HER2)^58^. By this means, cardiotoxicity was virtually abolished in hPSC-CMs^58^, and a Phase 1 clinical dose-escalation study confirmed the improvements expected on the basis of the pioneering human preclinical results^59^. No cardiac adverse events occurred in patients receiving this form of DOX alone, and the toxicity of combination therapy along with trastuzumab also was reduced^59^. Thus, human proof-of-principle was substantiated in hPSC-CMs (in this instance, cardiac safety of the targeted DOX), in advance of progression into human trials. Analogously, the cardiotoxicity of trastuzumab was found to be associated with defective energy metabolism in hPSC-CMs and was ameliorated by treatment with activators of AMP-activated protein kinase^55^, suggesting the potential for cardioprotection in this context by a current approved drug.

A further success in translational relevance is the progress made using hPSC-CMs for drug repurposing, in particular thus far where based on the cells’ fidelity to clinical phenotypes in certain hereditary heart disorders. This progress is perhaps most notable for personalized treatment of channelopathies such as long QT syndrome due to mutations in *KCNH2* that disrupt intracellular trafficking of the hERG potassium channel Kv11.1 (LQT2). Lumacaftor (the protein chaperone VX-809) was evaluated in patient-specific hPSC-CMs, and shown to shorten the cell culture equivalent of QTc, as measured with multi-electrode arrays. This benefit was achieved solely in patients with Class 2 mutations (which affect channel trafficking) but not in patients with Class 1 mutations (which affect channel synthesis)^60^. A clinically approved drug, Orkambi, combines Lumacaftor and Ivacaftor (a CFTR potentiator), rescues the analogous defect in patients with a homozygous *CFTR*-F508del mutation, and was taken forward into two patients with Class 2 mutations of LQT2, shortening QTc in both, as had been hypothesized^61^. Concomitantly, however, this landmark first-in-human study also acknowledged several “differences between the cellular model and clinical reality,” which provide instructive caveats, including the magnitude of rescue achieved (much greater in hPSC-CMs than in the clinic) and expression of hERG (higher in hPSC-CMs than in native adult CMs) ^61^.

What about cardiac drug development, more broadly than just reformulation or repurposing? Miniaturization of phenotypic assays to a 384-well format makes it possible to implement high-throughput chemical or genetic screens (HTS) for target validation and drug discovery, more rooted in human cardiac biology than has been possible heretofore. In an early example of tool-building toward high-throughput studies, hPSC-CMs were implemented to model myocarditis due to coxsackievirus B3, using CVB3-luciferase as an easy bioluminescent readout of virus proliferation^62^. Antiviral drugs including interferon-β1 and ribavirin were shown to suppress virus production. In addition, mechanistic insights were captured in this human cardiomyocyte milieu: by microarray profiling, interferon-β1 was shown to activate a network of downstream anti-viral genes including *EIF2AK2*, encoding protein kinase R, an inhibitor of viral mRNA translation ^62^.

For cardiac muscle hypertrophy, one readily assayable readout is the induction of brain natriuretic peptide, a highly dynamic protein with especially strong diagnostic and prognostic significance in the clinical setting^63^. Its induction is triggered in hPSC-CMs by endothelin-1, much as in non-human models of pathological hypertrophy, and its detection by ELISA or high-content imaging can be multiplexed with other parameters such as increased cardiomyocyte size^64^. In an early pilot phenotypic screen, hPSC-CMs were provoked at a saturating concentration of endothelin-1, and were treated in quadruplicate at 10 concentrations with candidate inhibitors including the calcium channel blocker verapamil, a PI3K-mTOR inhibitor, BEZ-235, and a broad-spectrum histone deacetylase inhibitor^64^. A design feature worth noting was the use of serum-free fatty acid-supplemented media, to accelerate cardiomyocyte maturation^64^. More fundamentally, these experiments demonstrated that inhibitor profiling in this human platform was robust and workable at scale.

## map4k4 AS A druggable target in human cardiac muscle cell death: target validation and compound development in HPSC-CMS

Many logical targets have failed in clinical trials for heart disease, perhaps especially those aiming to enhance cardiac muscle cell survival after myocardial infarction^10–15^. While specific shortcomings in trial design or implementation are sometimes culpable, what these failed trials have in common uniformly is that none was based on proof of efficacy in human preclinical studies, before proceeding into the clinic. The use of hPSC-CMs toward drug discovery for cardioprotection was championed in our recent study creating novel inhibitors of the stress-activated kinase MAP4K4 (mitogen-activated protein kinase kinase kinase kinase-4)^65^, an upstream member of the MAPK superfamily with connections to Jun N-terminal kinase^66,67^ and NFkB^68^, as well as to several non-canonical effectors as substrates^69,70^. The scientific case for MAP4K4 as a druggable target in heart muscle cell death began with human tissue characterization, finding that myocardial MAP4K4 was activated in end-stage heart failure regardless of cause (dilated, hypertrophic, ischemic, and anthracycline cardiomyopathy). MAP4K4 was likewise activated in a range of disease models in adult mouse myocardium and cultured rat cardiomyocytes, including ischemia/reperfusion injury and H_2_O_2_ as a surrogate oxidative stress. The causal role of MAP4K4 suggested by these observations was then corroborated using transgenic over-expression in mice, plus gain-of-function mutations, dominant-negative mutations, and gene silencing in rodent cardiomyocytes. Yet, even collectively, these methods—typical of the toolkit for academic target validation—leave altogether unanswered the question of whether MAP4K4 is a mechanistically sound therapeutic target in human heart muscle cell death.

Consequently, using hPSC-CMs as a human platform for target validation and proof-of-concept studies, the requirement for MAP4K4 in human cardiac cell death was affirmed by gene silencing, giving uniquely direct impetus to a small-molecule discovery program^65^. Throughout, several high-throughput platforms were utilized, including automated microscopy (DRAQ7 uptake, TUNEL staining for apoptosis), ATP generation, and cardiac troponin release. Protection was conferred in three wholly independent human cardiomyocyte lines, suggesting not only the reliability of hPSC-CMs as a model but also the unvarying dependence on MAP4K4 in the tested forms of cardiac cell death. Beyond these key readouts of viability, protective effects of inhibiting MAP4K4 were also proven under sublethal stress, using the Seahorse extracellular flux (XF) method to study mitochondrial function and FLIPR assays to measure calcium cycling. Cardiomyocyte viability and function (auxotonic force) were even preserved in human 3D engineered heart tissue^65^, a model with further maturity of structure and physiological properties^71^. Importantly, the pathway and compounds developed in hPSC-CMs were substantiated further by proof-of-concept studies in mice, with the MAP4K4 inhibitor reducing infarct size by more than 55% in blinded studies, even given an hour after injury^65^. These MAP4K4 studies demonstrate the pivotal role played by hPSC-CMs in validating a suspected target by gene silencing, then building a small-molecule program upon efficacy proven in this human platform.

Will treatments devised in human models be more likely to succeed than prior ones, upon eventual testing in the clinic? This overall hypothesis—the crux of using hPSC-CMs as a model in drug discovery—will require a decade or more to resolve empirically, as exemplars of this class progress into human trials. However, it is tantalizing to apply the “retrospectoscope”: examining the outcome, in human cardiomyocytes, of manipulating pathways whose success or failure is already known in reducing infarct size. Indeed, from this perspective, the potential predictive value of studies in hPSC-CMs is suggested by finding that human cardiac muscle cell death can be suppressed experimentally by β-adrenergic blockade (as found in METOCARD-CNIC^72,73^) but not by inhibiting p38 MAPK (as was ultimately true in SOLSTICE^14^).

## Preventing cardiac muscle cell death

By contrast to more reductionist but biased target-based approaches, phenotype-driven screens, discussed here and in the following sections, require no a priori assumption about the drug’s target, can pursue wholly novel or unanticipated mechanisms of action, engage the cell type-specific signaling and transcriptional context, and encompass diverse endogenous readouts of the disease^74^—including cardiomyocyte survival, but also myocyte function and creation.

In a mechanistically agnostic version of the MAP4K4 cell death studies detailed above, an unbiased chemical biology screen of nearly 50,000 small molecules was performed to identify and validate compounds that protect hPSC-CMs from H_2_O_2_^75^. Cardiomyocyte protection was demonstrated using ATP content as the primary end-point and cellular impedance as the secondary readout, a measure of monolayer integrity and contractile function. From this screen, in 1536-well format, using a 35% improvement in viability as the criterion, 220 hits were identified of which half were confirmed upon retesting. A novel compound, designated cardioprotectant-312, was found to protect hPSC-CMs from the oxidative stress of H_2_O_2_, triggering upregulation of the essential endogenous anti-oxidant, heme oxygenase-1^75^. Of note, no protection was seen in rat H9c2 cells, embryonic heart-derived myoblasts that are commonly used in toxicology research; conversely, none of the authors’ earlier lead compounds identified in H9c2 cells^76^ was protective in human cardiomyocytes. Together, these vivid reciprocal disparities highlight the importance of implementing human preclinical cardiac models, in lieu of basing human trials solely on results obtained in rodent cardiomyocytes alone.

## Attenuating diabetic cardiomyopathy

The power of hPSC-CMs for drug discovery to alleviate heart failure was demonstrated in two studies using patient-specific iPSCs to model diabetic cardiomyopathy^57,77^. To mimic the diabetic-like environment, normal hPSCs were cultured in a maturation medium to promote the substrate utilization of adult ventricular myocytes (fatty acid β-oxidation), then were subjected to glucose excess, plus endothelin-1 and cortisol^77^, mimicking the systemic environment. The combined effect was marked induction of brain natriuretic peptide (BNP), other molecular markers of cardiac hypertrophy, and myocyte enlargement itself. Functional abnormalities included less frequent Ca^2+^ transients, decreased beat amplitude, and increased beat irregularity. Lipid accumulation and peroxidation were other associated findings. The cardiomyopathic phenotype was captured, too, in cardiomyocytes derived from diabetic patient-specific iPSCs, even in the absence of the diabetic milieu ^77^.

This model was then used as a phenotypic drug screening platform, determining the success of a therapeutic compound as monitored by BNP production, nuclear area, and sarcomere organization (α-actinin staining). From a library of 480 compounds, 47 were identified that improved all three of these disease parameters^77^. These compounds encompassed diverse modes of action, including regulators of Ca^2+^ homeostasis (fluspirilene, thapsigargin, and the calmodulin inhibitor W7), Na^+^ and K^+^ channel blockers, phosphodiesterase inhibitors, and multiple protein kinase inhibitors (H89, K252a, SB202190). From this screen, in turn, compounds were found that rescued the reduction of Ca^2+^ transients in cardiomyocytes subjected to the diabetic milieu and improved the phenotype of cardiomyocytes from diabetic patients. Analogous improvement of diabetic phenotypes in hPSC-CMs were elicited by empagliflozin, an inhibitor of sodium-glucose co-transporters that are upregulated in diabetes, possibly explaining the unexpectedly improved cardiovascular mortality in trials of this compound for glycemic control^78^. Together, these findings support the utility of hPSC-CMs as models not merely for simple monogenic disorders, on the one hand, and for wild-type cardiomyocytes’ responses to lethal stress, on the other, but even for diabetic cardiomyopathy, a highly complex polygenic disease.

## Driving cardiac muscle cell proliferation

Ultimately, myocardial infarction can be viewed as a “myocyte-deficiency disease” whose phenotype is determined not just by the extent of myocyte death but also by the lack of functionally significant restorative growth. Indeed, the plausible clinical benefits of hPSC-CMs very clearly include therapeutic grafting, as cell therapy^35,79,80^, Recently, though, advances in understanding the fundamental biology of cardiac growth arrest have pointed to greater plasticity that was formerly evocable, with significant potential for restarting the cardiac cell cycle therapeutically, at least in model organisms^81,82^. Might induced proliferation as a route to heart repair also be amenable to exploration or triage in hPSC-CMs? As one starting point, functional screening of more than 10,000 hPSC-CM organoids was undertaken to optimize diverse aspects of the culture milieu, resulting in enhanced maturation and recapitulation of the adult heart’s notorious resistance to cell cycling, driven by a shift to fatty acid oxidation^83^. Conversely, this successful model of implementing cardiac cell cycle arrest in hPSC-CMs was then subjected to a functional screen of 105 small molecules, resulting in the identification of novel cell cycle activators, working through the mevalonate pathway^84^. Thus, notwithstanding the potential immaturity of pluripotent cell-derived myocytes with respect to cell cycle control, a post-mitotic phenotype could be imposed experimentally, and means to override it discovered.

## Challenges and prospects

The research advances reviewed here highlight the many benefits of hPSC-CMs as a transformative human platform for cardiac drug discovery^74^—accessible, scalable, faithful by a large number of clinically relevant parameters, amenable to genetic engineering, amenable to tissue engineering, able to capture patient variations, predictive of clinical success at least retrospectively, and predictive prospectively at least of success in whole-animal studies (Fig. 2; Table 1). One must, of course, temper optimism with caution. For the moment, it remains a supposition that new drugs developed by this route will achieve clinical success more reliably than drugs lacking human preclinical proof of effect. But, this hypothesis is testable—even if needing years of accumulated experience to do—while drawing ample credence from the established precedents and practices in cancer chemotherapy.

How might the predictive power of hPSC-CMs be augmented or ensured? Despite the predictive power shown even with routine 2D models, it is clear that existing lines—or, more accurately, their current embodiment in tissue culture—do not suffice to model all possible phenotypes of concern. The many acknowledged shortcomings, which have been mitigated to date only partially, include morphology (lack of sarcomere organization, T-tubules, and the normal mitochondrial density), molecular profile (weak expression of maturation-associated genes and splicing isoforms), metabolism (glycolysis, not fatty acid oxidation), contractility (lower maximum contractile force), and electrophysiology (lesser action potential upstroke velocity and amplitude)^48–51,85–95^. For instance, by comparison to adult human hearts, commercially available hPSC-CMs were uniformly deficient in the expression of KCNJ2, with higher than normal expression of HCN4 and large line-to-line variations in the other ion channels and membrane transporters assayed (CACNA1C, KCNH2, KCNQ1, SLC8A1, ATP1A1, ATP2A2)^51^. As a consequence, some pharmacological and pathobiological responses can be deficient or anomalous. In some reports, hPSC-CMs do not mirror the TdP risk of drugs with late sodium current effects, like ranolazine^32^, and hiPSC-CMs were less sensitive to hypoxia/reoxygenation than to other death signals^65,96,97^. Such disparities must be taken into account, whether inherent short-comings or idiosyncratic.

Efforts to enhance (further) the stem cell-derived myocytes’ predictive value center on manipulating chamber and cell sub-type specificity on the one hand, and on improving structural and functional maturity on the other. Purely pharmacological efforts at enhancing maturation in routine 2D culture include fatty acids^85,86^, thyroid hormone^87^, and inhibition of mTOR^88^. In another approach, transduction of the defectively expressed gene KCNJ2 has been applied to rescue the channel levels and promote aspects of fidelity directly^98^. More generally, however, the procedures of most proven value to enhance the maturity of hPSC-CMs include the use of 3D human engineered heart tissue (EHT), mechanical or electrical conditioning, and heart-on-chip technologies, advances discussed at length elsewhere^89–95^. The tissue engineering solutions to create more heart-like phenotypes in hPSC-CMs range in complexity from micropatterned 2D substrates to scaffolds, organoids, microfluidics, 3D bioprinting, and even the construction of hollow spheres. Apart from just geometry, key elements of these tissue engineering strategies notably include cyclic electrical or mechanical stimulation. Incorporation of other cell types can promote maturity or function, as well as the microvascularization required for oxygen delivery at larger scale than mere diffusion can confer. Indeed, 3D spheroids composed of hPSC-CMs plus hPDSC-derived endothelial cells showed progressive changes in gene expression typical of post-natal development^99^. Analogously, 3D culture of hPSC-CMs as engineered heart tissue in concert with cardiac fibroblasts, combined with long-term electrical stimulation, enables the development of physiological responses that are absent from the cardiomyocytes cultured routinely^93,100^. The reported adult-like properties included a positive force-frequency relationship, postrest potentiation of force, and inotropic beta-adrenergic responses to isoproterenol and dobutamine, as well as other compounds and pathways tested ^93,100^.

Although these collective efforts should be viewed with enthusiasm, progress toward standardization is confounded by the diversity of available hPSC lines, stem cell and differentiation media, physical substrata, timing, purification methods, presence or absence of serum, cell density (syncytial sheets, versus sporadic single cells), the heterogenous mixtures of cell types (atrial, ventricular, pacemaker cells, non-cardiomyocytes), and even lot-to-lot variation in ostensibly standardized cells. However, though procedures exist for the selective production of ventricular myocytes versus atrial myocytes or conduction system cells^93,101–104^, even these simple advances are not yet exploited uniformly. Ultimately, it is plausible that the drivers of standardization will include the emergence of consensus best practice solutions, but also adherence within the scientific community to procedures validated by multi-site initiatives such as CiPA, JiCSA, and InPulse, functioning as exemplars.

A complementary approach—obvious once the issue is raised—is also to improve the breadth of human cardiomyocytes surveyed, including but not limited to demographic features like gender and ethnic background. To illustrate, genetic determinants of susceptibility include a polymorphism in *ALDH2* that predominates in East Asians and renders the carriers’ myocytes much more vulnerable to ischemic heart damage^105^. Analogously, screening a single commercial line failed to capture the known cardiotoxicity of rosiglitazone, an anti-diabetic drug that enhances PPARγ activity but can exacerbate heart failure^106^. Indeed, marked inter-patient variation was later found in the response of hPSC-CMs to rosiglitazone, including adverse effects on reactive oxygen and nitrogen species, associated with divergent transcriptomic signatures that relate to NRF2-mediated oxidative stress^31^. A third demographic axis, aging, may be resistant to capture in hPSC-derived models, given the rejuvenation signature imparted by reprogramming to a primitive, pluripotent state; this aspect might be addressable using directly induced cardiomyocytes, instead^107,108^, for which a precedent is the success using forward programming to model age-related neurodegeneration^109^. Much like trials in the real world, clinical trials in a dish may need to take patient recruitment into account—along with their choices of compound, regimen, and readout—in developing robust new counter-measures to combat human heart disease.
